# 40 years of anorexia nervosa: why we should never give up

**DOI:** 10.1186/2050-2974-2-S1-O10

**Published:** 2014-11-24

**Authors:** Stephanie Hill, Anthea Fursland

**Affiliations:** 1 Centre for Clinical Interventions, Perth, Australia

## 

A case will be presented of a 54 year old woman who struggled with anorexia since age 16 without treatment, until a starvation fuelled incident of shoplifting triggered her to seek help for her eating disorder. Her anxious demeanour over her many phone calls and her intial BMI of 15 did not look hopeful. However, she has done remarkably well with 34 sessions of Enhanced Cognitive Behaviour Therapy over 15 months, and has stabilised at a BMI of 20.2, with an EDE-Q score within community norms. Creating a life without anorexia has been an ongoing challenge, but she is grateful that she is now physically and emotionally well enough to embark on this journey. This case illustrates why we should never give up on severe and enduring cases, and why we should always consider the possibility of full recovery, over and above quality of life improvements.

This abstract was presented in the **Treatment in Community and Inpatient Settings** stream of the 2014 ANZAED Conference.

